# Use of Hotels as a Disposition Alternative to Hospital Admission for Undomiciled Patients Undergoing SARS-CoV-2 Testing

**DOI:** 10.5811/westjem.57639

**Published:** 2023-05-03

**Authors:** Lucia C. Lin, Brendan B. McIntyre, Kaitlin McIntyre, Edward Castillo, Rachna Subramony, Allyson Kreshak

**Affiliations:** * University of California San Diego School of Medicine, San Diego, California; † University of San Diego, Department of Emergency Medicine, San Diego, California

## Abstract

**Introduction:**

The coronavirus 2019 (COVID-19) pandemic has presented various unprecedented challenges to healthcare systems globally, prompting society to adopt new preventative strategies to curb spread of the disease. Those experiencing homelessness have been particularly impacted because of barriers to practicing social distancing, inability to isolate, and poor access to care. Project Roomkey was established in California as a statewide measure to provide non-congregate shelter options for individuals experiencing homelessness to properly quarantine. On goal in this study was to analyze the effectiveness of hotel rooms as a safe disposition alternative to hospital admission for patients experiencing homelessness and who were also positive for severe acute respiratory syndrome coronavirus 2 (SARS-CoV-2).

**Methods:**

This was a retrospective, observational study that included chart review of patients who were discharged to the hotel from March 2020–December 2021. We recorded demographic information, index visit details, number of emergency department (ED) visits both a month prior to and following the index visit, admission rates, and number of deaths.

**Results:**

During this 21-month study period, a total of 2,015 patients who identified as undomiciled were tested for SARS-COV-2 in the ED for various reasons. Of those patients, 83 were discharged from the ED to the hotel. Of the 83 patients, 40 (48.2%) ultimately tested positive for SARS-CoV-2 during their index visit. Two patients returned to the ED within seven days with COVID-19-related symptoms, and 10 patients within 30 days. Two patients required subsequent admission with COVID-19 pneumonia. No deaths were recorded within the 30-day follow-up period.

**Conclusion:**

The availability of a hotel served as a safe alternative to hospital admission for patients experiencing homelessness and who were either suspected or confirmed to have COVID-19. It is reasonable to consider similar measures in the management of other transmissible diseases for patients experiencing homelessness who require isolation.

## INTRODUCTION

The coronavirus 2019 (COVID-19 pandemic caused by severe acute respiratory syndrome coronavirus-2 (SARS COV-2) presented various unprecedented challenges to healthcare systems globally since it was first identified in December 2019. According to the National Center for Health Statistics, the highly contagious viral illness had resulted in greater than 6.2 million deaths worldwide by May 2022, thereby quickly emerging as the worst global health crisis since the 1918 influenza pandemic.^1^ More than one million of those deaths occurred in the United States alone. Although significant progress in clinical research has led to better understanding of the disease and its subsequent management, the continued spread and emergence of variants are of increasing concern.^2^ Hospitals around the world continue to be overwhelmed by admissions due to COVID-19, prompting society as a whole to adopt new strategies on preventive measures to help curb the spread of the disease.

Those experiencing homelessness have been particularly impacted because of barriers to practicing social distancing, inability to isolate, and poor access to care. Due to these numerous barriers, more extensive interdisciplinary work has been required for individuals experiencing homelessness during the pandemic with emphasis on disposition planning.^3^ As of 2018, 47% of individuals experiencing homelessness in the US were living in California, and 69% of California’s homeless population was determined to be unsheltered (ie, they were living on the streets as opposed to emergency shelters or transitional housing. That number continues to climb.^4^

In a statewide attempt to address the COVID-19 pandemic, Project Roomkey was established in California in March 2020. The initiative aimed to provide non-congregate shelter options for individuals experiencing homelessness to properly quarantine, which would consequently aid in recovery, prevent further spread, and minimize strain on the healthcare system due to the disease. People eligible for Project Roomkey included those experiencing homelessness who had tested positive for SARS-CoV-2, had been exposed to the virus, or were at “high risk” of health complications should they have become infected including those who were elderly, immunocompromised, or had other medical comorbidities.^5^,^6^

In this study our goal was to analyze the effectiveness of hotel rooms as a safe quarantine option for undomiciled patients discharged from the emergency department (ED) during the COVID-19 pandemic from March 2020–December 2021.

## METHODS

### Study Design and Setting

This was a retrospective, observational study performed at an ED from an inner-city, tertiary care, teaching hospital in Southern California. We collected data from March 2020–December 2021 for a total of 21 months. A hotel in geographic proximity to our hospital that was funded by the state of California was designated as an alternative admission site for patients experiencing homelessness who were suspected or confirmed to be positive for SARS-CoV-2 and determined to have a low likelihood of needing further medical interventions in an inpatient setting.

**Population Health Research Capsule**

What do we already know about this issue?

*Those experiencing homelessness during the COVID-19 pandemic experienced barriers to practicing social distancing and accessing care*.

What was the research question?

*Are hotel rooms a safe disposition alternative to hospital admission for undomiciled patients with COVID-19?*

What was the major finding of the study?

*Two of 83 undomiciled patients (2.4%) who were discharged to a hotel were later admitted for pneumonia. No deaths were reported*.

How does this improve population health?

*Hotel rooms can be a safe disposition alternative for undomiciled patients with or suspected to have transmissible diseases, such as COVID-19*.

### Patients and Data Collection

Inclusion criteria for the study patients were as follows: 1) identified as homeless in the electronic health record according to information provided during registration; 2) tested positive for SARS-CoV-2 during their ED visit and/or were suspected to be SARS-CoV-2 positive while pending testing results based on symptoms including fever, cough, fatigue, anosmia, and ageusia; and 3) were medically stable for discharge. Excluded from the study were patients who were unable to perform activities of daily living and those exhibiting behavioral health issues that deemed them unsafe for hotel room solidarity.

Once eligibility was confirmed, social workers in the ED made arrangements to have the patient transported to one specific, state-funded hotel. At the hotel a registered nurse was on duty. The nurse was available to assist with acute medical needs, thereby potentially mitigating the need for a return visit to the ED from the hotel. The criteria for safe release from the hotel varied as the pandemic progressed and were based on information issued by the US Centers for Disease Control and in accord with the California Department of Public Health criteria and San Diego County guidelines.

Demographic information was collected including gender, race, and age. Other data points obtained included Emergency Severity Index (ESI), chief complaint, SARS-CoV-2 testing results, comorbidities, and disposition. The ESI is a five-level triage algorithm that has been shown to help facilitate reliable acuity assessment and predict patient disposition in the ED. We also recorded visits to the ED in the month both prior to and following the index visit and admission rates, as well as number of deaths.

### Statistical Methods

Given the small numbers of patients in the study, we analyzed the data using descriptive statistics.

## RESULTS

During this 21-month study period, a total of 2,015 patients who identified as undomiciled were tested for SARS-CoV-2 in the ED for various reasons. Of those patients, 83 were discharged from the ED to the hotel for quarantine purposes. Within this cohort of patients discharged to the designated hotel, 56 patients (67.5%) were male, 34 (41.0%) were White, 17 (20.2%) were Black, and the average age was 45.7 years (Table 1). The majority of patients had an ESI score of 3–4 (97.6%). Of the 83 patients, 40 (48.2%) ultimately tested positive for SARS-CoV-2 during their index visit, with one patient having an unknown result from a pending test ordered from an outside hospital. The most common presenting symptom was cough in 42 patients (50.6%), followed by 24 with fever (28.9%), 21 with shortness of breath (25.3%), and 18 with prior COVID-19 diagnosis and/or personal request for COVID-19 testing (21.7%). Common comorbidities represented in this cohort included 28 patients with hypertension (33.7%), 27 with psychiatric illness (32.5%), and 16 with chronic obstructive pulmonary disease and/or asthma (19.3%) (Table 2).

Following the index ED visit, five patients (6.0%) returned within seven days, of whom two presented with possible COVID-19-related symptoms, specifically worsening shortness of breath and hypotension. Nineteen of the 83 patients (22.9%) returned within 30 days, of whom 10 presented with possible COVID-19-related symptoms including new or worsening cough, shortness of breath, fever, and generalized weakness. Of those 19 patients returning within 30 days, three required hospital admission. Two of the three patients—the same patients who presented to the ED with COVID-related symptoms within seven days following their index visits—were hospitalized for COVID-19 pneumonia, while one was hospitalized for seizures secondary to alcohol withdrawal. No deaths were reported in the 30 days following ED discharge to the hotel (Table 3).

## DISCUSSION

With the high transmissibility of SARS-CoV-2, physical isolation of patients positive for this virus has been essential to mitigate spread.^7^ The implementation of an alternative to hospital admission, such as sequestering in hotel rooms for those experiencing homelessness amid a pandemic, was a novel intervention to facilitate the physical isolation of a population with limited resources to do so. This study demonstrated the feasibility and safety of securing disposition to a hotel for appropriately selected patients infected or concerned to be infected with SARS-CoV-2 and who were experiencing homelessness.

Leveraging the availability of an alternative, safe disposition from the ED for a population experiencing homelessness has several advantages. In the absence of this option, these patients would otherwise have required hospitalization for isolation. Hospitalization is not without risks, such as falls, delirium, and nosocomial infections including SARS-CoV-2.^8^ Thus, by avoiding unnecessary hospitalization, these risks may have been averted. Additionally, disposition of these patients to a hotel helped to mitigate hospital crowding due to the COVID-19 pandemic. Hospital crowding is characterized by a shortage of inpatient beds rather than lack of ED capacity, as had been previously suspected.^9^ Various factors can contribute to hospital crowding including increasing age of patients, hospital regulations, and the use of EDs as an alternative to primary care.^10^ With increased admissions and hospital crowding, the ED can transition from a temporary holding area to an extended patient care unit, thereby decreasing capacity for new admissions. As a result, delays in treatment, increased mortality, and a greater number of hospital readmissions may occur.^11^ Furthermore, recent studies have shown that when intensive care unit (ICU) beds reach capacity, the risk of death for patients infected with SARS-CoV-2 nearly doubled.^10^ One study found that once ICU occupancy reaches 85%, the chance of a COVID-19 patient dying was nearly 20% higher compared to when occupancy was between 45–85%.^12^

Medical safety is essential for patient disposition from the ED to a hotel. We did not establish firm medical criteria to guide patients’ medical appropriateness for the hotel; rather, we let the emergency physicians make this determination. If the patient was medically safe for discharge, then the patient was considered appropriate for disposition to the hotel. Interestingly, most of our patients had an ESI score of 3 or higher, suggesting overall medical stability.^13^ We did set behavioral standards and establish basic functionality regarding activities of daily living so that the patients would be functional and safe in the hotel. This decision-making process proved to be effective as we had a low rate of return to the ED, with just two of the 83 patients requiring subsequent hospitalization for COVID-19-related disease. Additionally, there were no deaths within 30 days of the index visit.

As of December 2020, Project Roomkey had provided hotel rooms for more than 22,000 people, which equated to about 8% of California’s homeless population and slightly over 10% of the state’s unsheltered population. In response to the success of Project Roomkey as a short-term emergency measure, Project Homekey was developed in a statewide effort to sustain and rapidly expand housing for individuals experiencing or at risk of homelessness^14^. The initiative had varied effects over different counties statewide, providing rooms to about 3% of the homeless population in San Diego County compared to 20% in San Francisco County. While our study notably included a smaller cohort, its conclusions are in alignment with more robust studies, such as that by Fleming et al, which demonstrated that shelter-in-place hotels with embedded health services may be an effective strategy to mitigate the risk of SARS-CoV-2 infection and reduce acute care use among undomiciled patients with a history of high health services use.^15^

Although established as a direct response to the COVID-19 pandemic, the use of hotel rooms could theoretically be expanded beyond the current pandemic in the management of various other communicable diseases. Repurposed hotel rooms can be differentiated from other shelters, which have stricter admission requirements and limitations from ineligibility criteria as well as limited availability. Furthermore, permanent shelters and temporary shelter alternatives, such as convention centers, entail congregate settings that can be counteractive when combating contagious illnesses. Theoretically, the use of hotel rooms could be considered in future public health interventions for transmissible disease outbreaks, such as tuberculosis, measles, scabies, meningitis, shingles, hepatitis A, and influenza. Notably, there is a paucity of literature demonstrating the effectiveness of hotel rooms in disposition-planning for communicable diseases other than COVID-19, as this is an area of emerging research.

## LIMITATIONS

There are several limitations to this study, including the small sample size and short period of observation. An additional limitation is that patients self-reported their status of experiencing homelessness. If a patient did not report that they were experiencing homelessness, then that patient would not have been included in this study. In this regard, the self-reporting status of experiencing homelessness may have limited selection and biased the study. Furthermore, we did not have access to the hotel nursing notes or have the potential to mitigate return visits to the ED from the hotel. Our inability to track hotel nurse check-ins with the patients while they were staying at the hotel is a limitation of the study. Additionally, as this was a single-center study with strong social support available to its ED patients, these results may not be generalizable to all EDs.

## CONCLUSION

The availability of a hotel for undomiciled patients presenting to the ED for quarantine purposes served as a feasible and safe alternative to hospital admission during the COVID-19 pandemic. It is reasonable to consider similar measures in the management of other transmissible diseases for patients experiencing homelessness and requiring isolation.

## Figures and Tables

**Table 1:** Demographics of 83 homeless patients who sheltered in a hotel during the COVID-19 pandemic.

	(N = 83)
Mean age	45.7
Gender	
Male	56 (67.5%)
Female	27 (32.5%)
Race	
White	34 (41.0%)
Black	17 (20.2%)

**Table 2:** Presenting symptoms and comorbidities at emergency department index visit.

	(N = 83)
Presenting Symptom	
Cough	42 (50.6%)
Fever	24 (28.9%)
Shortness of breath	21 (25.3%)
Prior COVID-19 diagnosis and/or personal request for COVID-19 testing	18 (21.7%)
Common Comorbidities	
Hypertension	28 (33.7%)
Psychiatric illness	27 (32.5%)
COPD and/or asthma	16 (19.3%)

**Table 3:** Summary of results.

	(N = 83)
SARS-CoV-2 testing during index visit	
Positive	40 (48.2%)
Negative	43 (51.8%)
Following Index ED visit	
Returned to ED within 7 days of index visit	5 (6.0%)
Returned to ED within 7 days AND required hospitalization	2 (2.4%)
Returned to ED within 30 days of index visit	19 (22.9%)
Returned to ED within 30 days AND required hospitalization	3 (3.6%)
Deaths within 30 days	0 (0%)

*SARS-CoV-2*, severe acute respiratory syndrome coronavirus 2; *ED*, emergency department.
